# Single versus double tendon transfer for improving shoulder function in brachial plexus birth palsy: a meta-analysis of comparative studies

**DOI:** 10.1186/s12891-025-08803-9

**Published:** 2025-06-03

**Authors:** Ahmed O. Sabry, Mohamed K. A. Genedy, Yara Kassem, Marwa El-Difrawy, Reem Shalata, Maziad Hennidi, Farah A. R. Salama, Badr Ali Mohammed Badr, Mohamed Abdel-Wahed

**Affiliations:** 1 Department of Orthopedic Surgery, Cairo University, El Saray Street, Manial - Cairo, 11956 Egypt; 2 Faculty of Medicine, Cairo University, Cairo, Egypt

**Keywords:** Tendon Transfer, Teres Major, Latissimus dorsi, OBPP, NBPP

## Abstract

**Background:**

Obstetric brachial plexus palsy (OBPP) may result in lifelong shoulder dysfunction despite nerve repair surgery. Tendon transfer has emerged as a surgical option to restore external rotation and abduction in OBPP sequelae. Single and double tendon transfer techniques have been developed, but the optimal approach remains debated. This meta-analysis is the first to compare the range of motion outcomes and complications, particularly loss of midline function (LOM), between the two techniques in managing OBPP sequelae.

**Methods:**

On January 2025, systematic literature search was performed in five databases (PubMed, Cochrane Library, Embase, Scopus and Web of Science), to identify studies comparing single and double tendon transfer in children with OBPP. Eligible studies underwent quality assessment via MINORS criteria and a meta-analysis using RevMan was conducted to compare the functional outcomes and complications.

**Results:**

Five retrospective studies encompassing 189 patients were analyzed. The pooled mean difference (MD) in the total modified Mallet score between groups was statistically insignificant (*MD = 0.09; 95% CI= -0.68 to 0.85;p* > 0.05). Analyses for the sub-scores revealed no differences across all sub-scores. However, for Latissimus Dorsi (LD) single transfers, Abdel-Ghani et al. reported 7.7-fold greater odds of LOM (*OR = 0.13; 95% CI = 0.04–0.42;p* < 0.05), whereas the pooled LOM of Teres Major (TM) single transfers were not significantly different (*OR = 0.40; 95% CI = 0.11–1.47;p* > 0.05).

**Conclusion:**

In conclusion, our meta-analysis suggests that both single and double tendon transfers achieve comparable functional outcomes. However, the data suggests that LD single tendon transfer potentially lowers rate of LOM, though further research is needed to confirm this finding.

**Clinical trial number:**

Not applicable.

**Supplementary Information:**

The online version contains supplementary material available at 10.1186/s12891-025-08803-9.

## Introduction

Obstetric brachial plexus palsy (OBPP) is a relatively common perinatal injury, with an incidence ranging from 0.1 to 8.1 per 1,000 live births globally; these rates have remained relatively the same over the past 50 years despite advances in maternal and fetal care [1, 2]. It’s primarily maternal, fetal, and obstetric factors where traction forces applied on the brachial plexus during delivery, particularly in shoulder dystocia, which is the most frequently implicated risk factor, alongside macrosomia, prolonged labor, operative deliveries, multiparity, pregestational and gestational diabetes, and oxytocin use [3–5].

In cases with mild delay or weakness, conservative management and follow-up are often sufficient, while more severe injuries typically require early nerve surgery to restore function [6]. Despite early treatment, many patients may continue to experience persistent deficits, including limited shoulder abduction and external rotation, as well as impairments in elbow and hand function. These residual deficits can compromise overall functional outcomes and quality of life, with studies reporting lasting impairments in 20–33% of children at age of 10–12 [1, 6, 7].

For patients who do not achieve sufficient recovery after primary nerve reconstruction, tendon transfer surgery serves as a secondary procedure to improve shoulder function in OBPP. First described by L’Episcopo in the 20th century, this technique aims to restore external rotation and abduction of the shoulder by redistributing muscle forces [8–10]. First, the operation involved the transfer of the Teres Major (TM) tendon to the posterior humerus at the triceps level, repurposing it as an external rotator. Subsequent modifications by Zachary and Hoffer incorporated the Latissimus Dorsi (LD) tendon, which led to the use of the double tendon transfer technique [8, 11, 12]. This approach was based on the premise that transferring two tendons would provide greater force to improve the efficacy of shoulder abduction and external rotation despite the suggestion that it may compromise midline function [8, 11, 12]. This possible limitation led to the re-attention to the LD or TM single tendon transfer procedure as a way to decrease midline deficits while achieving similar abduction and external rotation, which made the decision between both options debatable [9, 13].

The aim of this systematic review and meta-analysis was to compare the efficacy of single and double tendon transfer techniques in treating OBPP sequelae by focusing on functional outcomes in the form of the modified Mallet score and its sub-scores in addition to complications to determine the most suitable technique for managing this condition.

## Methodology

### Protocol registration

This systematic review and meta-analysis was designed to compare the outcomes of single versus double tendon transfers in children with OBPP-related sequelae of shoulder deficits, followed the PRISMA guidelines and the Cochrane Handbook for Interventions [14, 15], and was registered on PROSPERO with the identifier number (CRD42024612046).

### Data sources & search strategy

On the 21st of January 2025, we conducted a comprehensive search of five databases (PubMed, Embase, the Cochrane Library, the Web of Science Core Collection, and Scopus) for clinical studies that compared single and double tendon transfers in children with OBPP, with single and double tendon transfers nested and combined by Boolean operators, as described in Additional File 1. Table S1. In addition, we cross-referenced the relevant text.

### Eligibility criteria

We included studies involving pediatric population suffering range of motion (ROM) dysfunction as a sequela of OBPP who underwent single or double tendon transfer procedures, specifically LD and/or TM transfers, with follow-up for functional outcomes and complications. Studies were excluded if they were noncomparative or involved patients with nerve injuries outside the C5–T1 nerve roots.

### Study selection

The Rayyan web-based application was used to conduct screening. The search results were imported, and duplicates were manually removed. Two authors independently assessed the titles and abstracts. Next, full-text articles were collected for a second, blind screening via the same method. Any conflicts in the screening process were resolved through discussion.

### Data extraction

The authors collected quantitative and qualitative data for each outcome from the included studies. The extracted data were separated into three standardized Excel spreadsheets [16]. The first and second sheets detail the study and population characteristics, such as the study ID, design, age, sex, type of operation, injury level, and side. The third sheet included outcomes, which included validated, and routinely used tools for assessing shoulder function in children with OBBP such as the modified Mallet score [17], and Gilbert score [18], as well as complications, including contractures and loss of midline function (LOM), defined as the inability to touch the belly.

### Quality assessment

Two independent authors evaluated the included papers via the Methodological Index for Non-Randomized Papers (MINORS) criteria [19]. Twelve methodological categories were assessed, and each domain was graded on a scale of zero to two, with zero indicating no reporting, one indicating inadequate reporting, and two indicating adequate reporting.

### Statistical analysis

A meta-analysis was conducted to compare the treatment effects of single and double tendon transfers. The data were analysed via Review Manager (RevMan) software (version 5.4.1; Cochrane) [20]. The groups were compared via mean differences (MDs) and odds ratios (ORs) with 95% confidence intervals (CIs) via a random effects model. The outcomes analysed included the postoperative total modified Mallet score, its sub-scores, and complications. Studies were weighted via the inverse variance method, and a *p* value of ≤ 0.05 was considered statistically significant for the pooled effect size. Heterogeneity across studies was assessed via Cochran’s Q test and the I² statistic, which was categorized as follows, Low (*I*^*2*^ *< 25%*), moderate (*I*^*2*^ *= 25–75%*), and high (*I*^*2*^ *> 75%*) [21], with a *p* value of *≤ 0.05*, indicate significantly heterogeneous studies. Sensitivity analysis via leave-one-out meta-analysis was conducted via OpenMetaAnalyst [22] to assess the effect of each study on the overall pooled effect.

To facilitate pooling, Abd El-Ghani et al.’s reported Gilbert score was rescaled into the corresponding modified Mallet sub-scores, as shown in the supplementary material (Additional file 1. Table S2) [23].

The variance was zero for some outcomes (Abdel-Ghani 2012, Abzug 2020, Topley 2021) and was missed in certain studies (Abzug 2022, Greenhill 2019). Abzug et al. [9] and Greenhill et al. [24] The standard deviations (SDs) were estimated as follows. The t value was derived from the p value, and the standard error (SE) of the difference was calculated via the formula SE = mean difference ÷ t value. The average standard deviation for each group was subsequently determined from the SE of the difference via the formula $\:SDaverage\:=\:\surd\:1\:n1\:+\:1\:n$ [25, 26]. When the SD was zero in any of the included studies, the analysis was conducted twice. Once with the SD imputed as 0.0001 and once with the zero-variance study excluded. The results from both analyses were then compared to evaluate the impact of studies with zero variance.

## Results

### Search results and study selection

**Fig. 1:**
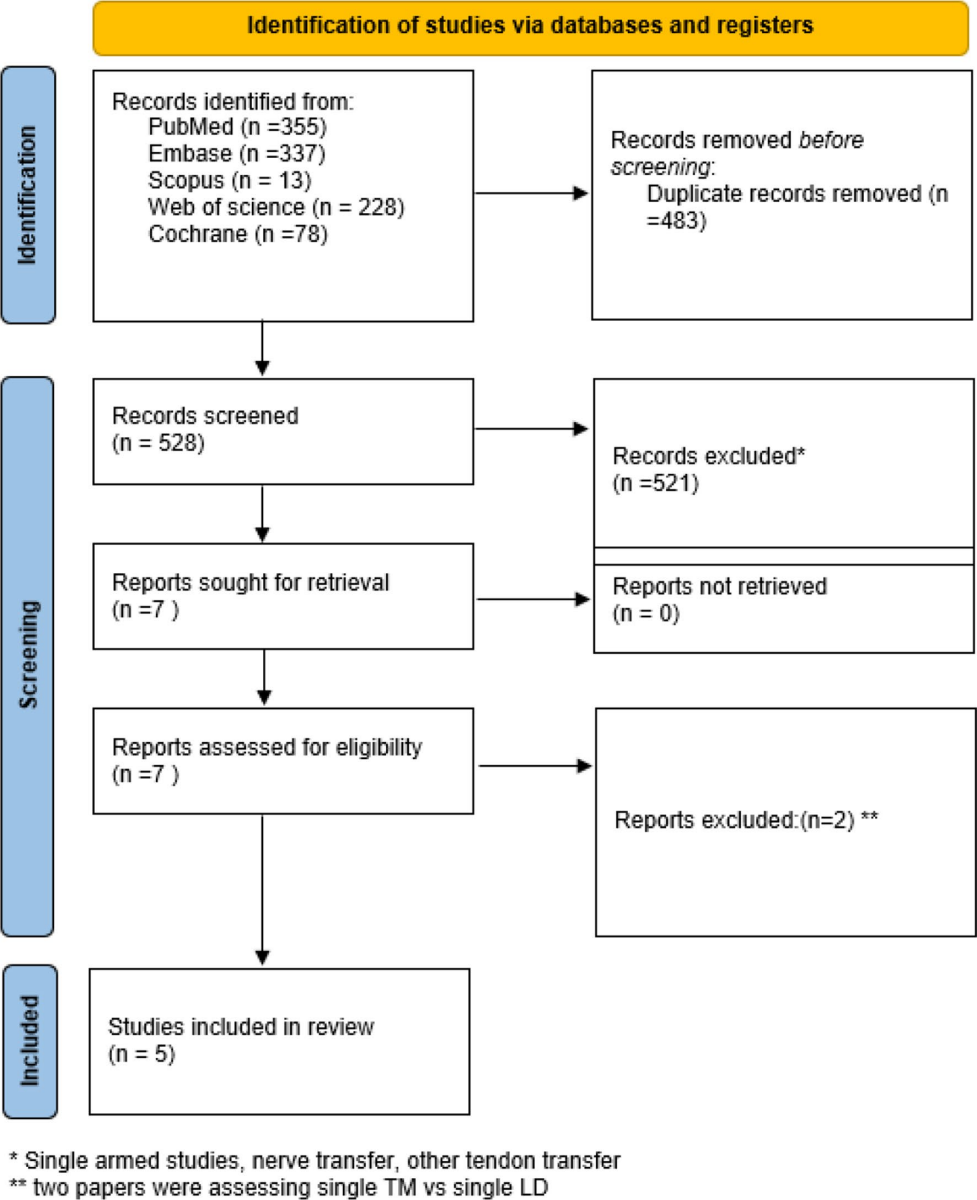
Prisma flow chart

**Fig. 2:**
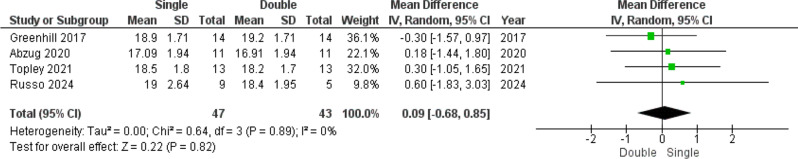
Shows the comparison of the total modified Mallet scores between the single and double tendon groups. The meta-analysis revealed no significant difference between the two techniques (*MD = 0.09; 95% CI = -0.68–0.85;p* > 0.05), with no significant heterogeneity (*I² = 0%;p* > 0.05)

The search across five databases yielded 1,011 records. After 483 duplicates were removed, 528 records remained for screening. Of these, 521 studies were excluded because they were single-arm studies involving nonbirth trauma cases (e.g., cadavers or adults) or focused on trapezius tendon transfer. Seven full-text articles were assessed for eligibility, all of which were successfully retrieved. Two studies were excluded for not evaluating double tendon transfer, resulting in five studies deemed eligible for inclusion (Figs. 1, 2).

### Characteristics of the included studies

All included studies [9, 18, 24, 27, 28] were retrospective cohort studies with mean follow-up periods ranging from 0.43 to 4.1 years. However, Russo et al. [28] was a mixed retrospective-prospective case series in which patients were initially reviewed retrospectively and those meeting eligibility criteria underwent additional prospective follow-up.

The majority of studies were conducted in the United States and utilized the TM muscle for single tendon transfer, with the modified Mallet scale used as the grading system. In contrast, Abdel-Ghani H et al. [18], in Egypt, used the LD tendon and the modified Gilbert shoulder grading system [9, 18, 24, 27, 28] (Table 1).

**Table 1:** Summary characteristics of the included studies

Author, Country, Journal & Year of Publication	Study Type/Level of Evidence	Single muscle transfer	Associated Routine Soft Tissue Release	Grading System Used for Assessment	Follow-up period (Mean ± SD) years
Abdel-Ghani H et al., Egypt, The Egyptian Orthopaedic Journal [2011]	Retrospective Cohort Study	Latissimus Dorsi	Subscapularis sliding	Modified Gilbert Shoulder Grading System	1.62 ± 0.95 years
DA Greenhill et al., USA, Journal of Pediatric Orthopedics [2017]	Retrospective Cohort Study	Teres Major	Shoulder reduction, nerve transfer/graft (1year prior to tendon transfer)	Modified Mallet scale	4.1 ± 3.1 years
Abzug JM et al., USA, HAND [2020]	Retrospective Cohort Study	Teres Major	NR	Modified Mallet scale	0.43 ± 0.19 years
Topley, Matthew T. et al., USA, Journal of Hand Surgery [2021]	Retrospective Cohort Study	Teres Major	Subscapularis Release/ Elevation, Prior Botox and closed reduction, Previous nerve grafting	Modified Mallet scale	4 ± 1.3 years
Russo, Stephanie A. et al., USA, Journal of Shoulder and Elbow Surgery [2024]	Mixed Retrospective/Prospective Case Series	Teres Major	Concomitant joint release/reduction, Cricoidectomy, Subscapularis release, Pectoralis major, lengthening, Previous nerve transfer	Modified Mallet scale	0.9 ± 4.8 years

NR: Not Reported

The included studies [9, 18, 24, 27, 28] included between 15 and 63 patients each, with a total of 154 if we excluded the nonmatched cohorts of Greenhill et al. and 189 if we included them. The mean age at surgery ranged from 1.8 to 6.6 years (single tendon transfer). 1.9–7.4 years; double tendon transfer. 1.6–5.14 years). A total of 151 patients and 116 patients, if we excluded the nonmatched cohorts of Greenhill et al., had C5–6 nerve root involvement, whereas 33 had C5–7 injuries, and 5 had C5–T1 involvement (Table 2).

**Table 2:** Summary characteristics of population

Author, Country, Journal & Year of Publication	Total Patients Included (*n*)	Patients per Operation Group (*n*)		Total Shoulders Involved (*n*)		Injury Level (*n*)			Age at Operation (Mean ± SD) years	Age at Operation According to Group	
		Single	Double	Left	Right	C5-6	C5-7	C5-T1		Single	Double
Abdel-Ghani H et al., Egypt, The Egyptian Orthopaedic Journal [2011]	63 [34 M, 29 F]	18	45	23	40	51	8	4	3.56 ± 1.72	3.65 years	3.68 years
DA Greenhill et al., USA, Journal of Pediatric Orthopedics [2017]	28 [11 M, 17 F + unmatched 19 M, 16 F]	14	14 (49)*	N/A	N/A	28 (63)*	0	0	2.2 ± 1.07 (3.0 ± 1.4) years*	1.9 ± 0.7 years	2.5 ± 1.3 (3.3 ± 1.4) years*
Abzug JM et al., USA, HAND [2020]	22 [12 M, 10 F]	11	11	N/A	N/A	0	22	0	3.8 ± 2.5 years	3.6 ± 1.38 years	4.1 ± 3.34 years
Topley, Matthew T. et al., USA, Journal of Hand Surgery [2021]	26 [8 M, 18 F]	13	13	13	13	26	0	0	1.8 ± 1.2 years	2.0 ± 1.3 years	1.6 ± 1.1 years
Russo, Stephanie A. et al., USA, Journal of Shoulder and Elbow Surgery [2024]	15**	10	5	N/A	N/A	11	3	1	6.6 ± 4.3 years	7.4 ± 5 years	5.14 ± 1.46 years

n: Number, M: Male, F: Female

*In Greenhill et al. the results of 49 patients were reported in the double tendon transfer, but only 14 were included in the matched cohort

**Russo didn’t provide information on the number of males and females

### Quality assessment

The quality assessment of the included studies [9, 18, 24, 27, 28] via the MINORS criteria yielded total scores ranging from 19 to 20 out of a maximum of 24. All studies [9, 18, 24, 27, 28] demonstrated adequate performance in most domains, but consistent limitations were observed across studies [9, 18, 24, 27, 28] in some domains (inclusion of consecutive patients, unbiased assessment of prospective collection of data, and calculation of study size). These limitations are inherited from the retrospective design of the included studies [9, 18, 24, 27, 28]. For the baseline equivalence domain, most studies [9, 24, 27] employed the matched groups design, except Abdel-Ghani et al. [18] (2012) and Russo et al. (2024) [28]^,^ which had imbalanced sample sizes **(**Table 3**).**

**Table 3:** Summary of minors quality assessment

Study Id	A clearly stated aim	Inclusion of consecutive patients	Prospective collection of data	Endpoints appropriate to the aim of the study	Unbiased assessment of the study endpoint	Follow-up period appropriate to the aim of the study	Loss to follow-up less than 5%	Prospective calculation of the study size	An adequate control group	Contemporary groups	Baseline equivalence of groups	Adequate statistical analyses	Total score
Abdel-Ghani et al., 2012	2	2	1	2	2	2	1	1	2	2	0	2	19
Greenhill et al., 2017	2	2	1	2	2	2	1	1	2	2	2	2	21
Abzug et al., 2020	2	2	1	2	2	2	1	1	2	2	2	2	21
Topley et al., 2021	2	2	1	2	2	2	1	1	2	2	2	2	21
Russo et al., 2024	2	2	1	2	2	2	1	1	2	2	0	2	19

## Meta-analysis results

### Modified mallet score and sub-scores

The analysis of modified Mallet sub-scores revealed no significant differences between the single and double tendon transfer groups, as detailed in Supplementary File 1 (Additional File 2. Figures S1–S10). Heterogeneity was generally low across all sub-scores, except for the internal rotation subscore, which demonstrated moderate heterogeneity regardless of inclusion or exclusion zero-variance studies (*I² = 61%;p* = 0.05 and I² = 72%; *p* < 0.05) (Additional File 2. Figures S5 and S6). However, leave-one-out sensitivity analysis revealed no difference regardless of the removed study (Additional File 2. Figure S7).

### Complications

**Fig. 3:**
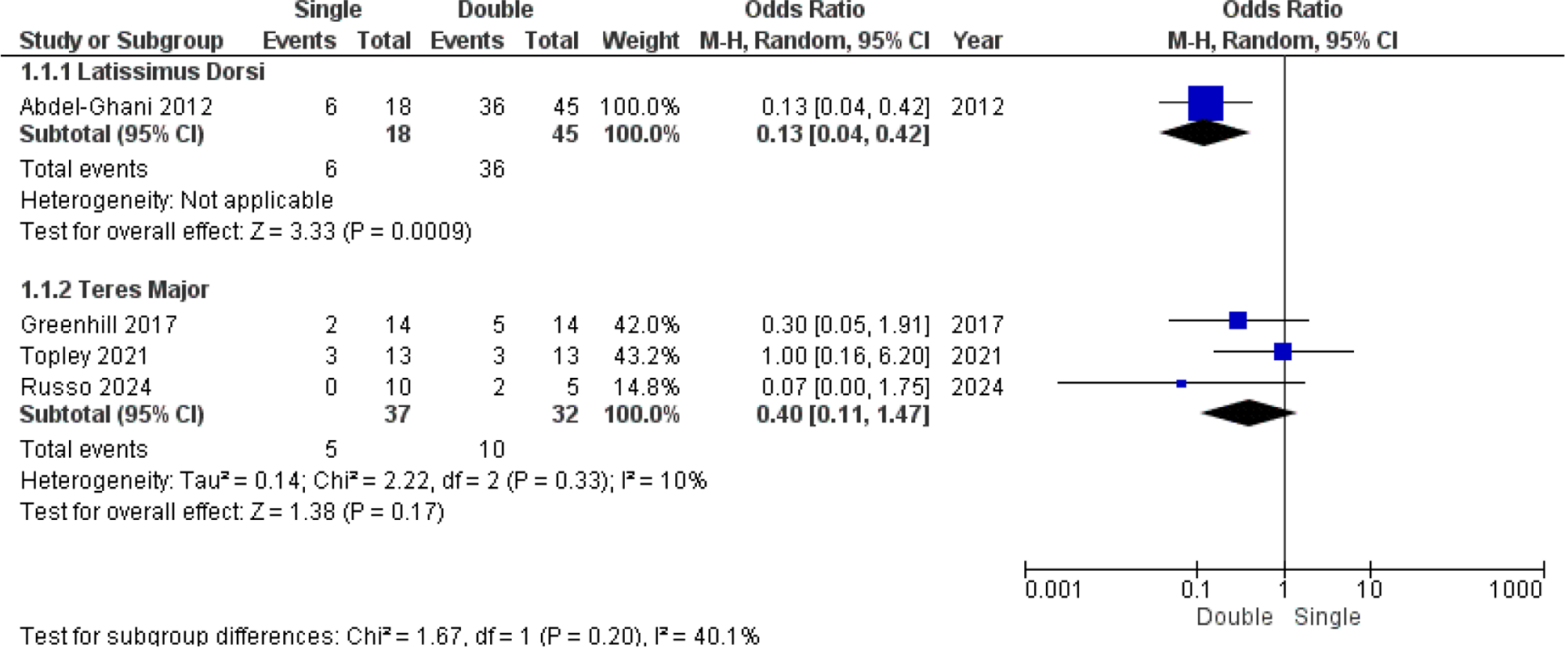
Loss of midline function forest plot

LOM was the most frequently reported complication, occurring in 57 of 132 patients (43%). It was predominantly reported in Abdelghani et al.‘s report, affecting 42 of 63 total patients (66%). Although Abdel-Ghani et al. reported a high rate of LOM in general, their study demonstrated that the odds of LOM being 7.7-fold (*OR = 0.13*,* 95% CI. 0.04 to 0.42*, *p* < 0.05). Notably, the single tendon transfer in this study utilized the LD, whereas other studies primarily used the TM. The double-tendon group presented a slightly greater rate of LOM when compared to single-TM group, however the dichotomous analysis didn’t show statistically significant (*OR = 0.40; 95% CI. 0.11–1.47;p* > 0.05), with low heterogeneity (*I² = 10%*, *p* > 0.05). Tests for subgroup differences were nonsignificant (*p* > 0.05), indicating that the effect directions were comparable between the LD and TM subgroups (Fig. 3).

Abduction contracture was another notable complication (*4/132*,* 3%*) that was reported in only Russo et al., 2024 [28] and was predominantly observed in the double tendon transfer group (*3/77*,* 4%*) (Table 4).

**Table 4:** Summary of complications

Study Id	Single	Double
Abdel-Ghani et al., 2012	-6/18 (33%) Loss of Midline Function. -0/18 (0%) Intermittent shoulder subluxation.	-36/45 (80%) Loss of Midline Function. -1/45 (2.2%) Intermittent shoulder subluxation.
Greenhill et al., 2017*	-2/14 (14.3%) Loss of Midline Function.	-5/14 (35.7%) Loss of Midline Function.
Abzug et al., 2020	NR	NR
Topley et al., 2021	-3/13 (23%) Loss of Midline Function.	-3/13 (23%) Loss of Midline Function.
Russo et al., 2024	-0/10 (0%) Loss of Midline Function. -1/10 (10%) abduction Contracture by at least 10% in a neutral position.	-2/5 (40%) Loss of Midline Function -3/5 (60%) abduction Contracture by at least 10% in a neutral position

NR: Not Reported

*For Greenhill et al. we compared the complication rates in the matched cohort only

## Discussion

When primary initial surgical attempts fail to reestablish adequate muscle function and balance in OBPP patients, it often results in structural abnormalities such as abnormal humeral head positioning against the posterior glenoid, posterior subluxation, dislocation, and deformities like glenoid dysplasia, retroversion, and humeral head flattening, which necessitates shoulder tendon transfer surgery is indicated. It aims to restore shoulder abduction and external rotation while simultaneously minimizing complications [9, 24]. Historically, double tendon transfer was believed to provide superior over single tendon transfer in functional recovery by enhancing external rotation and abduction [11]. This meta-analysis is the first to compare both tendon transfers techniques in OBPP shoulder sequelae.

Our analysis revealed no significant difference in the postoperative modified Mallet score or its subscores when comparing the double versus single tendon transfer groups, Consequently, minimizing LOM should be a priority when determining the surgical strategy, as LOM can adversely affect a child’s daily activities—including self-care, writing, and overall upper limb coordination [28–30]. LOM was the most frequently reported complication, with a higher incidence noted in the double tendon transfer cohort, particularly Abdel-Ghani et al. observed the highest rate of LOM, a finding potentially attributable to the routine execution of subscapularis sliding in both study groups. This maneuverer may disrupt the balance between the internal and external rotator muscles, thereby increasing the overall risk of LOM. Notably, when comparing TM tendon transfers with double tendon transfers, the double tendon group exhibited a marginally greater rate of LOM, although the associated odds ratio did not reach statistical significance. Further insights were provided by Abdelaziz et al. and Ibrahim et al. studies, which directly examined single tendon transfers using either the TM or LD [31, 32]. Ibrahim et al. reported an LOM incidence of 50% in TM single transfer cases, compared with 35% in those undergoing LD single transfer; however, this inter-group difference was not statistically significant [32]. Similarly, AbdelAziz et al. documented a higher rate of loss of internal rotation in the TM group than in the LD group, though this observation was likewise not supported by statistical testing [31].

Considering that single tendon transfer appears to achieve comparable functional outcomes and similar odds of LOM—with the added benefit of a less complex surgical procedure—it may represent the preferred approach in many cases; particularly, LD single tendon transfer, which potentially advantageous in reducing LOM when compared directly and indirectly with TM single tendon transfer [31, 32]. However, given the limited evidence currently available, this recommendation should be weighed against individual patient factors such as preoperative shoulder function, donor muscle availability, and the surgeon’s technical expertise.

Post-operative care, including early physical therapy and splinting can help maintain ROM, minimize contractures, and optimize functional recovery, while in complex anatomical challenges, including contractures, dislocations, and bony deformities Adjuvant interventions can be performed [24, 28, 29]. For instance, subscapularis sliding is frequently combined with LD and/or TM transfers to address internal rotation contractures, where releasing or lengthening the subscapularis tendon creates passive external rotation, balancing shoulder forces and reducing stiffness risks [18]. Similarly, glenohumeral joint reduction are often utilized to correct posterior dislocations caused by chronic muscle imbalance, often accompanied by anteroinferior capsular release to facilitate joint realignment before tendon transfer stabilizes the reduction [18, 27]. In severe contractures, additional releases of the pectoralis major or conjoined tendons may optimize exposure and external rotation, though these structures often lax postoperatively [18, 27]. For older patients with advanced deformity, a humeral rotational osteotomy can reposition the arm functionally by addressing malrotation, and in rare instances, adjuncts such as coracoidectomy or pectoralis major lengthening are used to relieve impingement or targeted contractures [28]. Ultimately, the choice of soft tissue, bony, or capsular procedures is tailored to the severity of contracture, reducibility of dislocation, patient age, and joint deformity, thereby synergizing with tendon transfers to restore shoulder balance, enhance active external rotation and abduction, and improve long-term outcomes [18, 27].

### Limitations and recommendations

Our review included only five retrospective studies which relied on the modified Mallet score which limited precision of our analysis because of the broad categorization of motor functions, not to mention relying on subjective visual assessment, and retrospective records which might have introduced bias [27].

In our review we emphasize the need for prospective randomized studies with larger cohorts to draw definitive conclusions. We also recommend the use of ROM and muscle strength or motion capture analysis if feasible to enable precise analysis.

## Conclusion

In conclusion, our meta-analysis suggests that both single and double tendon transfers achieve comparable functional outcomes. However, the data suggests that LD single tendon transfer potentially lowers rate of LOM, though further research is needed to confirm this finding.

## Electronic supplementary material

Below is the link to the electronic supplementary material.

Supplementary Material 1

Supplementary Material 2

## Data Availability

The data that support the findings of this study are available within the manuscript.

## References

[1] Van der Looven R, Le Roy L, Tanghe E, Samijn B, Roets E, Pauwels N, et al. Risk factors for neonatal brachial plexus palsy: a systematic review and meta-analysis. Dev Med Child Neurol. 2020;62:673–83. 31670385 10.1111/dmcn.14381

[2] Louden E, Marcotte M, Mehlman C, Lippert W, Huang B, Paulson A. Risk factors for brachial plexus birth injury. Child (Basel). 2018;5:46. 10.3390/children5040046 PMC5920392 29596309

[3] Dunbar DC, Vilensky JA, Suárez-Quian CA, Shen PY, Metaizeau J-P, Supakul N. Risk factors for neonatal brachial plexus palsy attributed to anatomy, physiology, and evolution. Clin Anat. 2021;34:884–98. 33904192 10.1002/ca.23739

[4] Johnson GJ, Denning S, Clark SL, Davidson C. Pathophysiologic origins of brachial plexus injury. Obstet Gynecol. 2020;136:725. 32925630 10.1097/AOG.0000000000004013

[5] Nihan Hande Akcakaya MSC. Evaluation of the Obstetrical Brachial Plexus Injuries with Forensic Perspective. 2021. https://hasekidergisi.com/articles/doi/haseki.galenos.2021.7332. Accessed 3 Jan 2025.

[6] Evidence that nerve. surgery improves functional outcome for obstetric brachial plexus injury - PubMed. https://pubmed.ncbi.nlm.nih.gov/32588706/. Accessed 3 Jan 2025.

[7] Annika J, Paul U, Anna-Lena L. Obstetric brachial plexus palsy - A prospective, population-based study of incidence, recovery and long-term residual impairment at 10 to 12 years of age. Eur J Paediatr Neurol. 2019;23:87–93. 30458977 10.1016/j.ejpn.2018.06.006

[8] Tendon transplantation in obstetrical paralysis - ScienceDirect. https://www.sciencedirect.com/science/article/abs/pii/S0002961034901434. Accessed 3 Jan 2025.

[9] Abzug JM, Miller E, Case AL, Hogarth DA, Zlotolow DA, Kozin SH. Single versus double tendon transfer to improve shoulder external rotation during the treatment of brachial plexus birth palsy. Volume 17. New York, N,Y): Hand; 2022. pp. 55–9. 10.1177/1558944720911211 PMC8721786 32188298

[10] Cavallaro D, Mikalef P, Power D. A comparison of tendon and nerve transfer surgery for reconstruction of upper limb paralysis. J Musculoskelet Surg Res. 2019;3:69.

[11] Zachary RB, TRANSPLANTATION OF TERES MAJOR AND LATISSIMUS DORSI FOR, LOSS OF EXTERNAL ROTATION AT SHOULDER. Lancet. 1947;250:757–8. 10.1016/s0140-6736(47)90763-0 20271222

[12] Hoffer MM, Wickenden R, Roper B. Brachial plexus birth palsies. Results of tendon transfers to the rotator cuff. J Bone Joint Surg Am. 1978;60:691–5. 681392

[13] Waters PM, Bae DS. Effect of tendon transfers and extra-articular soft-tissue balancing on glenohumeral development in brachial plexus birth palsy. J Bone Joint Surg Am. 2005;87:320–5. 15687154 10.2106/JBJS.C.01614

[14] Page MJ, McKenzie JE, Bossuyt PM, Boutron I, Hoffmann TC, Mulrow CD, et al. The PRISMA 2020 statement: an updated guideline for reporting systematic reviews. BMJ. 2021;372:n71. 33782057 10.1136/bmj.n71 PMC8005924

[15] Cochrane Handbook for Systematic Reviews of Interventions. https://training.cochrane.org/handbook. Accessed 26 Nov 2024.

[16] Free Online Spreadsheet Software. Excel| Microsoft 365. https://www.microsoft.com/en-us/microsoft-365/excel?msockid=3218872d75d360293968922b746a6109. Accessed 23 Nov 2024.

[17] Bae DS, Waters PM, Zurakowski D. Reliability of three classification systems measuring active motion in brachial plexus birth palsy. J Bone Joint Surg Am. 2003;85:1733–8. 12954832 10.2106/00004623-200309000-00012

[18] Abdel-Ghani H, Hamdy KA, Basha N, Tarraf YN. Tendon transfer for treatment of internal rotation contracture of the shoulder in brachial plexus birth palsy. J Hand Surg Eur Vol. 2012;37:781–6. 22736741 10.1177/1753193412451401

[19] Slim K, Nini E, Forestier D, Kwiatkowski F, Panis Y, Chipponi J. Methodological index for non-randomized studies (minors): development and validation of a new instrument. ANZ J Surg. 2003;73:712–6. 12956787 10.1046/j.1445-2197.2003.02748.x

[20] RevMan. Systematic review and meta-analysis tool for researchers worldwide| Cochrane RevMan. https://revman.cochrane.org/info. Accessed 23 Nov 2024.

[21] Higgins JPT, Thompson SG, Deeks JJ, Altman DG. Measuring inconsistency in meta-analyses. BMJ. 2003;327:557–60. 12958120 10.1136/bmj.327.7414.557 PMC192859

[22] OpenMeta-Analyst. open-source, cross-platform software for advanced meta-analysis| Cochrane Colloquium Abstracts. https://abstracts.cochrane.org/2010-keystone/openmeta-analyst-open-source-cross-platform-software-advanced-meta-analysis. Accessed 4 Jan 2025.

[23] Al-Qattan M, El-Sayed A. Obstetric brachial plexus palsy: the mallet grading system for shoulder Function—Revisited. Biomed Res Int. 2014;2014:398121. 24527447 10.1155/2014/398121 PMC3909974

[24] Greenhill DA, Smith WR, Ramsey FV, Kozin SH, Zlotolow DA. Double versus single tendon transfers to improve shoulder function in brachial plexus birth palsy. J Pediatr Orthop. 2019;39:328–34. 31169755 10.1097/BPO.0000000000000965

[25] Chi K-Y, Li M-Y, Chen C, Kang E, Cochrane Taiwan. Ten circumstances and solutions for finding the sample mean and standard deviation for meta-analysis. Syst Rev. 2023;12:62. 37005690 10.1186/s13643-023-02217-1 PMC10068165

[26] Approximating the Normal Tail Probability and its Inverse for Use on a Pocket Calculator| Journal of the Royal Statistical Society Series C. Applied Statistics| Oxford Academic. https://academic.oup.com/jrsssc/article-abstract/38/1/69/6985608. Accessed 4 Jan 2025.

[27] Topley MT, Russo SA, Chafetz RS, Zlotolow DA, Kozin SH, Richards JG. Scapulothoracic and Glenohumeral Contributions to Humerothoracic Kinematics in Single Versus Double Tendon Transfers in Patients With Brachial Plexus Birth Injury. J Hand Surg Am. 2022;47:897.e1-897.e9. 10.1016/j.jhsa.2021.06.026 34489135

[28] Russo SA, Nice EM, Chafetz RS, Richards JG, Zlotolow DA, Kozin SH. Impact of tendon transfer on scapulothoracic and glenohumeral motion in children with brachial plexus birth injuries. J Shoulder Elb Surg. 2024;:S1058-2746(24)00559-7. 10.1016/j.jse.2024.06.027 39151671

[29] Long-Term Results of Isolated Latissimus Dorsi to Rotator Cuff Transfer in Brachial Plexus Birth. Injury - PubMed. https://pubmed.ncbi.nlm.nih.gov/38868463/. Accessed 4 Jan 2025. 10.1055/s-0044-1786817 PMC11168807 38868463

[30] Abzug JM, Wyrick-Glover TO, Case AL, Zlotolow DA, Kozin SH. Loss of midline function in brachial plexus birth palsy patients. J Pediatr Orthop. 2019;39:e232–5. 30211803 10.1097/BPO.0000000000001251

[31] Abdelaziz AM, Abdelfath MA, Ismail MA, Wahd YESH, Ali AM, Akeed TA. Comparison of latissimus dorsi versus Teres major tendon transfer to restore external rotation of the shoulder in patients with Erb palsy. J Hand Surg Asian Pac Vol. 2025;30:55–62. 39676607 10.1142/S242483552550016X

[32] Ibrahim MR, Abdelmaksoud IM, Ahmad MH, Semaya AE. Comparing the results of latissimus dorsi versus Teres major transfer in children with obstetric brachial plexus injury and residual shoulder sequelae. Ann Plast Surg. 2023;90:144–50. 36688857 10.1097/SAP.0000000000003434

