# Supplementary material for: Single versus double tendon transfer for improving shoulder function in brachial plexus birth palsy: a meta-analysis of comparative studies
Source: BMC Musculoskelet Disord. 2025 Jun 3;26:554. doi: 10.1186/s12891-025-08803-9 (PMC12135586; doi:10.1186/s12891-025-08803-9)
Supplement: Supplementary file 1 — Supplementary Material 1 [file 12891_2025_8803_MOESM1_ESM.docx]

**Supplementary File 1**

**Title.** Single Versus Double Tendon Transfer for Improving Shoulder Function in Brachial Plexus Birth Palsy: Meta-Analysis of Comparative Studies

**Content:**

Table S1: Search strategy details

Table S2: Conversion from Gilbert Score to Modified Mallet Sub-Scores

Table S1: Search strategy details

21/1/2025

| **Database** | **Search Terms** | **Search Field** | **Search Results** |
| --- | --- | --- | --- |
| **PubMed** | (Obstetric OR Birth OR Neonatal OR infant OR pediatric OR child OR "shoulder dystocia") AND (Brachial OR Erb’s) AND (Latissimus OR "teres major" OR double OR single OR isolated OR combined) AND (tendon OR transfer OR reconstruction) | All Field | 355 |
| **Cochrane** | (Obstetric OR Birth OR Neonatal OR infant OR pediatric OR child OR "shoulder dystocia") AND (Brachial OR Erb’s) AND (Latissimus OR "teres major" OR double OR single OR isolated OR combined) AND (tendon OR transfer OR reconstruction) | All Field | 61 Reviews, 15 Trials, 2 protocols |
| **Scopus** | TITLE-ABS-KEY((Obstetric OR Birth OR Neonatal OR infant OR pediatric OR child OR "shoulder dystocia") AND (Brachial OR Erb's) AND (Latissimus OR trapezius OR "teres major" OR double OR single OR isolated OR combined) AND (tendon OR transfer OR reconstruction)) | Title, Abstract, Key words | 13 |
| **Web Of Science (core collection)** | (Obstetric OR Birth OR Neonatal OR infant OR pediatric OR child OR "shoulder dystocia") AND (Brachial OR Erb’s) AND (Latissimus OR "teres major" OR double OR single OR isolated OR combined) AND (tendon OR transfer OR reconstruction) | All Field | 228 |
| **Embase** | ((Obstetric or Birth or Neonatal or infant or pediatric or child or "shoulder dystocia") and (Brachial or Erb's) and (Latissimus or "teres major" or double or single or isolated or combined) and (tendon or transfer or reconstruction)).mp. [mp=title, abstract, heading word, drug trade name, original title, device manufacturer, drug manufacturer, device trade name, keyword heading word, floating subheading word, candidate term word] | All Field | 337 |

Table S2: Conversion from Gilbert Score to Modified Mallet Sub-Scores

| **Gilbert Score** | **Rescaled Modified Mallet Sub-Scores** | |
| --- | --- | --- |
|  | **Abduction** | **External Rotation** |
| Stage III: ≥90° abduction, with active external rotation (0-30°) | **Grade** IV (>90°) | **Grade** III (0-20°) |
| Stage IV: 90-120° abduction, with active external rotation (30-60°) | **Grade** IV (>90°) | **Grade** IV (>20°) |
| Stage Va: 120-150° abduction, with active external rotation (30-90°) | **Grade** IV (>90°) | **Grade** IV (>20°) |
| Stage Vb: >150° abduction, with active external rotation (>90°) | **Grade** IV (>90°) | **Grade** IV (>20°) |
