# Supplementary material for: Single versus double tendon transfer for improving shoulder function in brachial plexus birth palsy: a meta-analysis of comparative studies
Source: BMC Musculoskelet Disord. 2025 Jun 3;26:554. doi: 10.1186/s12891-025-08803-9 (PMC12135586; doi:10.1186/s12891-025-08803-9)
Supplement: Supplementary file 2 — Supplementary Material 2 [file 12891_2025_8803_MOESM2_ESM.docx]

**Supplementary File 2**

**Title.** Single versus Double Tendon Transfer for Improving Shoulder Function in Brachial Plexus Birth Palsy: A Meta-Analysis of Comparative Studies

**Content:**

Figure S1: Modified Mallet score (Abduction; studies with zero variance included)

Figure S2: Modified Mallet score (Abduction; studies with zero variance removed)

Figure S3: Modified Mallet score (External Rotation; studies with zero variance included)

Figure S4: Modified Mallet score (External Rotation; studies with zero variance removed)

Figure S5: Modified Mallet score (Internal Rotation; studies with zero variance included)

Figure S6: Modified Mallet score (Internal Rotation; studies with zero variance removed)

Figure S7: Modified Mallet score (Internal Rotation; leave-one-out sensitivity analysis)

Figure S8: Modified Mallet score (Hand to Spine)

Figure S9: Modified Mallet score (Hand to Mouth)

Figure S10: Modified Mallet score (Hand to Neck)

Figure S1: Modified Mallet score (Abduction; studies zero with variance included)
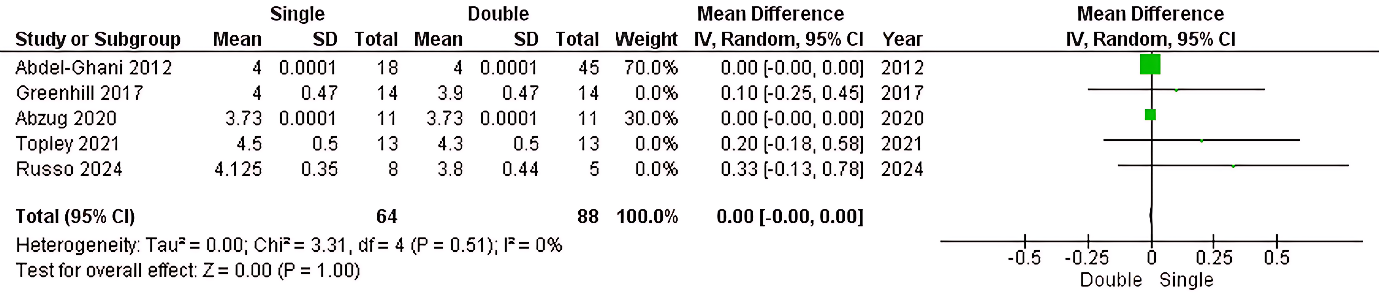


The results did not show any pooled results as the studies in which there were no MD between the groups, the variance was zero and it was replaced by 0.0001. The studies in the meta-analysis are weighted using inverse variance and as the variance is nearly zero then these studies had huge weights (one had 70% [Abdel-Ghani 2012] and the other is 30% [Abzug 2020]). Thus, the weight of the whole meta-analysis is taken by these studies, and these two studies did not show any difference (MD is zero), then there is no difference between the groups (pooled MD is zero).

Figure S2: Modified Mallet score (Abduction; studies with zero variance removed)
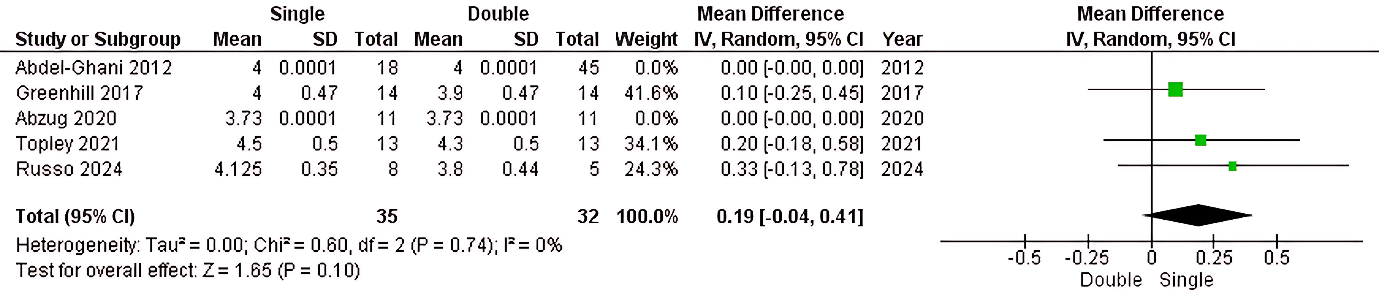


The results still showed no significant difference between the groups (n = 67; MD = 0.19; 95% CI = −0.04 to 0.41; p= 0.1), there was no heterogeneity across the studies (I^2^= 0%; p= 0.74).

Figure S3: Modified Mallet score (External Rotation; studies with zero variance included)
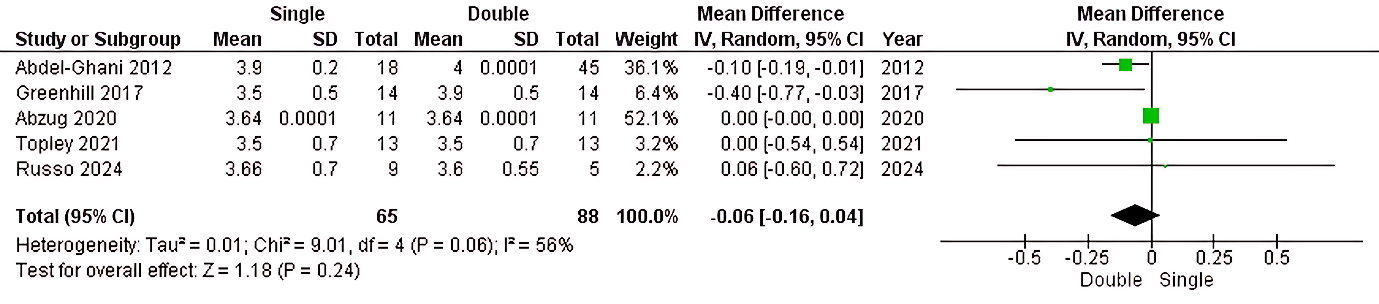


The results show no significant difference between the groups (n = 153; MD = −0.06; 95% CI = −0.16 to 0.04; p= 0.24), there was a no significant moderate heterogeneity across the studies (I^2^= 56%; p= 0.06). However, the two studies (Abdel-ghani 2012 and Abzug 2020) with no MD between the groups and zero variance accounts for 88.2% of the meta-analysis.

Figure S4: Modified Mallet score (External Rotation; studies with zero variance removed)
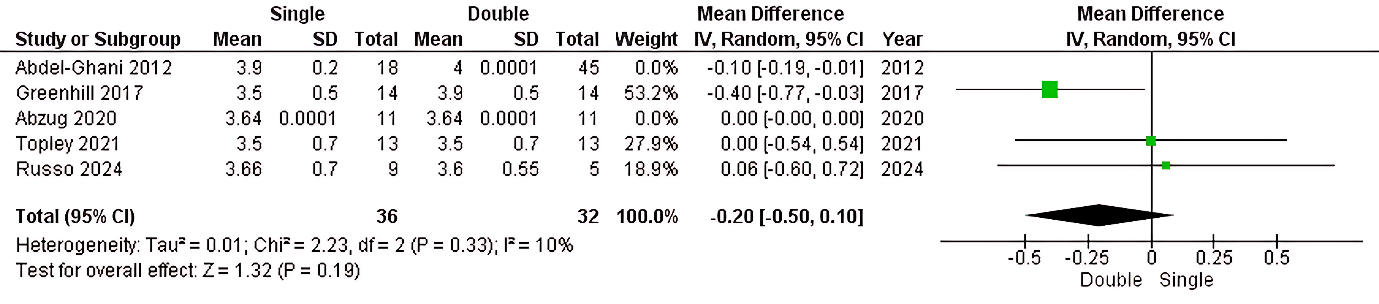


the results showed no significant difference between the groups (n = 68; MD = -0.2; 95% CI = −0.50 to 0.10; p= 0.19), there was no significant heterogeneity across the studies (I^2^= 10%; p= 0.33).

Figure S5: Modified Mallet score (Internal Rotation; studies with zero variance included)
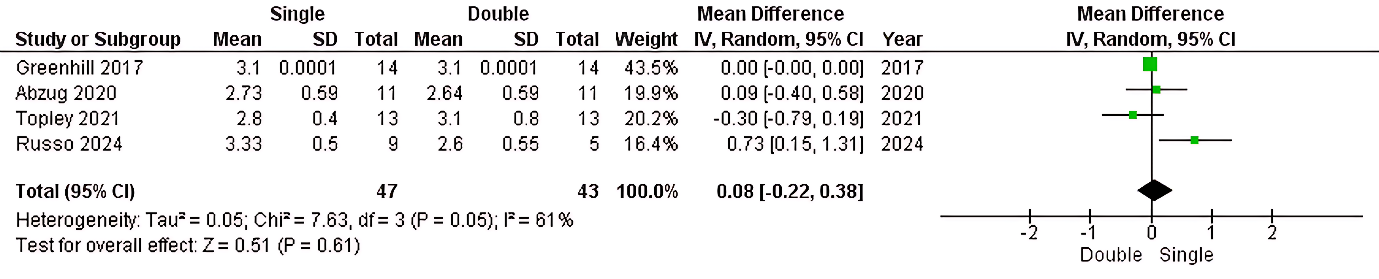


The results show no significant difference between the groups (n = 90; MD = 0.08; 95% CI = −0.22 to 0.38; p= 0.61), there was a significant moderate heterogeneity across the studies (I^2^= 61%; p= 0.05). However, one study of Greenhill 2017 with no MD between the groups and zero variance accounts for nearly 50% of the meta-analysis (43.5%).

Figure S6: Modified Mallet score (Internal Rotation; studies with zero variance removed)
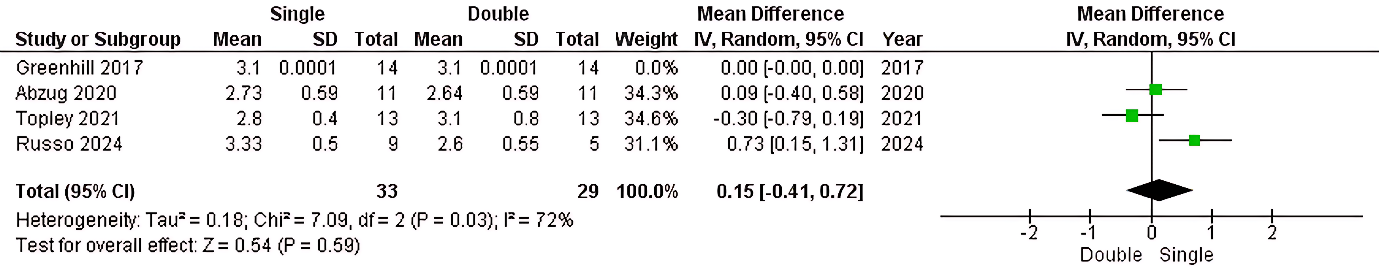


The results showed no significant difference between the groups (n = 62; MD = 0.15; 95% CI = −0.41 to -0.72; p= 0.59), there was significant moderate heterogeneity across the studies (I^2^= 72%; p= 0.03).

Figure S7: Modified Mallet score (Internal Rotation; leave-one-out sensitivity analysis)
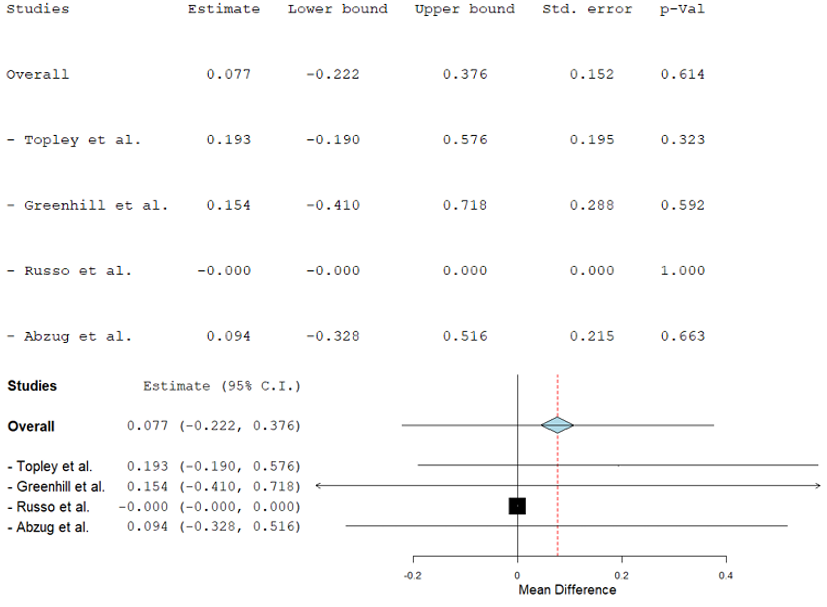


According to sensitivity analysis removing any of the included studies will not affect the overall pooled results of the meta-analysis.

Figure S8: Modified Mallet score (Hand to Spine; studies with zero variance included)
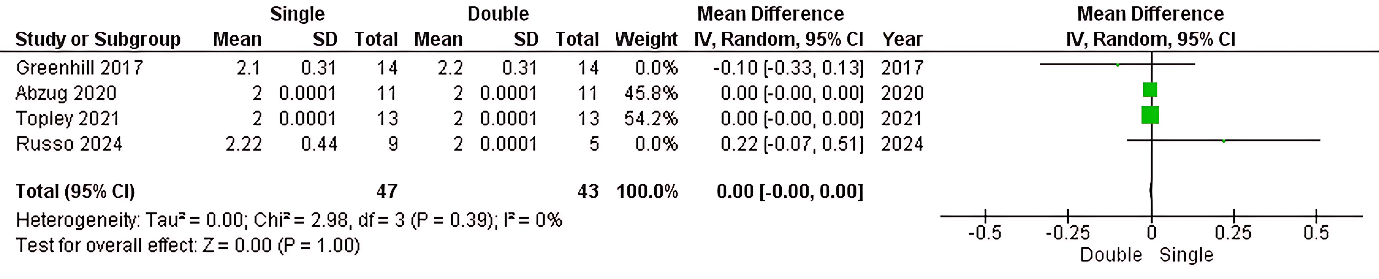


The results did not show any pooled results as the studies in which there were no MD between the groups, the variance was zero and it was replaced by 0.0001. These studies took the whole weight of the meta-analysis (one had 45.8% [Abzug 2020] and the other is 54.2% [Topley 2021]). Thus, these two studies did not show any difference (MD is zero), then there is no difference between the groups (pooled MD is zero).

Figure S9: Modified Mallet score (Hand to Mouth; studies with zero variance included)
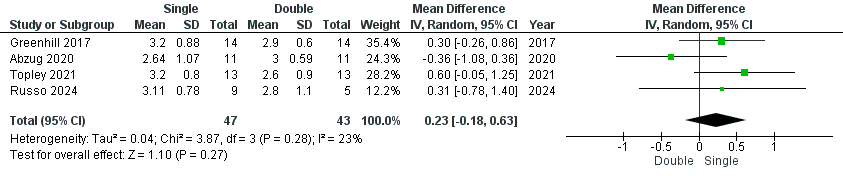


The results show no significant difference between the groups (n = 90; MD = 0.23; 95% CI = -0.18 to 0.63; p= 0.27), there was no significant heterogeneity across the studies (I^2^= 23%; p= 0.28).

Figure S10: Modified Mallet score (Hand to Neck)
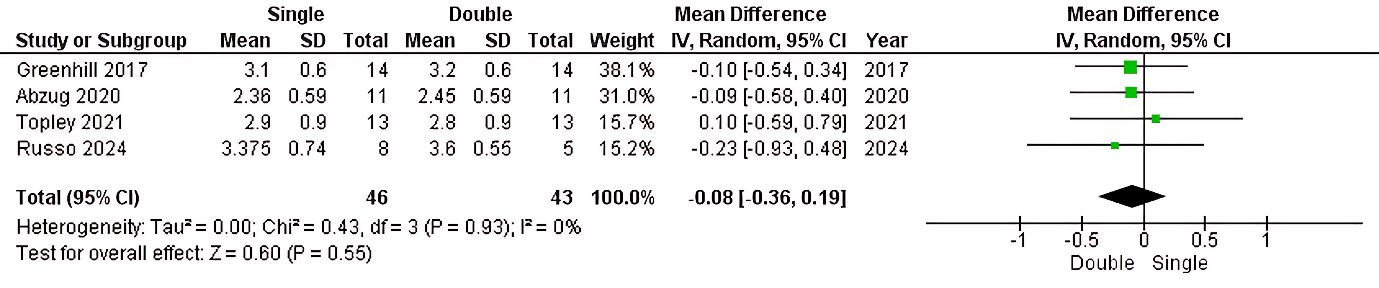


The results show no significant difference between the groups (n = 89; MD = -0.08; 95% CI = -0.36 to 0.19; P = 0.55), there was no heterogeneity across the studies (I^2^= 0%; p= 0.93).
